# Effectiveness of national evidence-based medicine competition in Taiwan

**DOI:** 10.1186/1472-6920-13-66

**Published:** 2013-05-07

**Authors:** Yi-Hao Weng, Ken N Kuo, Chun-Yuh Yang, Hsun-Hsiang Liao, Chiehfeng Chen, Heng-Lien Lo, Wui-Chiang Lee, Ya-Wen Chiu

**Affiliations:** 1 Department of Pediatrics, Chang Gung Memorial Hospital, Chang Gung University College of Medicine, Taipei, Taiwan; 2 Center for Evidence-Based Medicine, College of Medicine, Taipei Medical University, Taipei, Taiwan; 3 Division of Preventive Medicine and Health Services Research, Institute of Population Health Sciences, National Health Research Institutes, Miaoli, Taiwan; 4 Department of Public Health, Kaohsiung Medical University, Kaohsiung, Taiwan; 5 Taiwan Joint Commission on Hospital Accreditation, New Taipei, Taiwan; 6 Division of Plastic Surgery, Department of Surgery, Wan Fang Hospital, Taipei Medical University, Taipei, Taiwan; 7 Department of Public Health, School of Medicine, College of Medicine, Taipei Medical University, Taipei, Taiwan; 8 Institute of Hospital and Health Care Administration, National Yang-Ming University, Taipei, Taiwan; 9 Master Program in Global Health and Development, College of Public Health and Nutrition, Taipei Medical University, 250 Wu-Hsing Street, Taipei, 110, Taiwan; 10 Health Policy and Care Research Center, Taipei Medical University, Taipei, Taiwan

## Abstract

**Background:**

Competition and education are intimately related and can be combined in many ways. The role of competition in medical education of evidence-based medicine (EBM) has not been investigated. In order to enhance the dissemination and implementation of EBM in Taiwan, EBM competitions have been established among healthcare professionals. This study was to evaluate the impact of competition in EBM learning.

**Methods:**

The EBM competition used PICO (patient, intervention, comparison, and outcome) queries to examine participants’ skills in framing an answerable question, literature search, critical appraisal and clinical application among interdisciplinary teams. A structured questionnaire survey was conducted to investigate EBM among participants in the years of 2009 and 2011. Participants completed a baseline questionnaire survey at three months prior to the competition and finished the same questionnaire right after the competition.

**Results:**

Valid questionnaires were collected from 358 participants, included 162 physicians, 71 nurses, 101 pharmacists, and 24 other allied healthcare professionals. There were significant increases in participants’ knowledge of and skills in EBM (*p* < 0.001). Their barriers to literature searching and forming answerable questions significantly decreased (*p* < 0.01). Furthermore, there were significant increases in their access to the evidence-based retrieval databases, including the Cochrane Library (*p* < 0.001), MD Consult (*p* < 0.001), ProQuest (*p* < 0.001), UpToDate (*p* = 0.001), CINAHL (*p* = 0.001), and MicroMedex (*p* = 0.024).

**Conclusions:**

The current study demonstrates a method that successfully enhanced the knowledge of, skills in, and behavior of EBM. The data suggest competition using PICO queries may serve as an effective way to facilitate the learning of EBM.

## Background

Evidence-based medicine (EBM), a clinical practice consistent with the current best evidence, has been proposed as a core competence to help health professionals improve care quality [1-3]. It integrates clinical epidemiology, biostatistics, research methods, and informatics into health care. The process mainly involves four sequential steps: formulating a well-focused question based on a clinical problem, accessing and verifying relevant evidence from the literature, critical appraising the validity of the contemporary research, and applying the findings to clinical decision-making [4]. A clear answerable question is composed of four components – problem, intervention, comparison, and outcome – referred to by the acronym PICO [5,6]. In order to match the relevant scientific literatures and improve the retrieval of evidence, it is important to determine what is the problem of patient (P) first, then to what intervention (I) has been done, followed by comparing (C) the effect of different interventions, and finally measuring the outcome (O). PICO has been a major component of teaching evidence-based searching [7,8].

There are increasing examples illustrating that health professionals hold positive attitude toward EBM [9-12]. Nevertheless, their knowledge and skills pertaining to the implementation of EBM are relatively insufficient [12-15]. Many campaigns have been used to disseminate EBM into health professionals, mostly are organized lectured models, such as education, training programs, and workshops [16-21]. Yet, they often lack interdisciplinary interaction and relevance in their clinical settings. It is imperative for continuing education to maintain the active cognitive learning process in this respect.

Competition may offer an opportunity to attract all disciplines of healthcare professions by introducing the fun and excitement of learning. It has been used as an active tool for acquiring cognitive, effective psychomotor skills and knowledge. Education and competition are intimately related and can be combined in many ways. The role of competition in education has been investigated [22,23]. However, there is no general agreement as to what constitutes the best way of putting competitions to good use in educating EBM. Thus, the purpose of current study is to introduce a useful model of EBM competition and investigate its impact on the belief, knowledge, skill, barrier, and behavior of the healthcare professionals.

## Methods

### The National EBM competitions in Taiwan

Since 2006 Taiwan Joint Commission of Hospital Accreditation (TJCHA) introduced EBM competitions with collaboration of National Health Research Institutes (NHRI), and later joined with Taiwan Evidence-based Medicine Association (TEBMA) in an effort to transfer EBM into clinical practice. The competition was developed using information infrastructure and game-specific elements. There were two main phases for the contest: preparation and execution.

The initial phase involved preparing the entire framework: competition rules, competition tasks including scenario setting, and judging procedures. The rules are well formulated and transparent to avoid misleading participants (Table 1). The competition teams were classified into two levels – basic and advanced – by the ability of EBM implementation. Overall, the teams composed of non-experienced participants were categorized into basic groups. In contrast, the teams composed of repeat participants were categorized into advanced groups. In addition, the teams from awarded hospitals were requested to participate in the advanced groups.

**Table 1:** Rule of EBM competition

**Participants**	A cooperative team is composed of 3 healthcare personnel with at least two different professions (such as physicians, nurses, pharmacists and so on).
	■ Basic group
	■ Advanced group
**Task**	A. Each team has to develop at least two PICO questions according to the clinical scenario provided by the organizer at the inception of competition.
	B. Each team has to integrate all tasks above and submitted a Microsoft PowerPoint file at the end of competition within a total of 3 hours timeframe.
	C. Each team has10 minutes to present their task results in front of the judges and all participants.
	1. Each team has to state clearly detail strategies for searching answers based on the above PICO questions.
	2. Each team has to describe the tools applied in appraising selected articles, and their judgment according the criteria of their appraisal tool.
	3. Each team has to elaborate how the study conclusion can be implied to the patient in the clinical scenario and what should be considered.
**Grade weighting**	A. A total of five judges evaluate participating team performances according 5 domains:
	1. quality and quantity of PICO questions
	2. literature search
	3. critical appraisal
	4. clinical application
	5. presentation
	B. Under each domain, there are several sub-criteria. Each domain composes 20% of the total score. Final score of each team are the sum of 5 domain score.

PICO: problem, intervention, comparison, and outcome.

**Figure 1:**
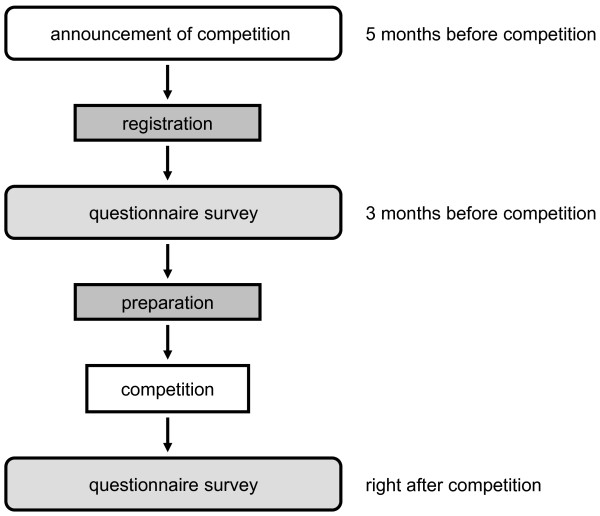
Scheme of study design.

Five months prior to the EBM competition, NHRI and TJCHA invited all hospitals and medical centers to organizing their healthcare professionals for participation by an official letter stating the details of the competition (Figure 1). Participants were organized as interdisciplinary teams and requested to register at three months or earlier prior to the EBM competition.

The second phase is the execution of actual competition. Participants were requested to determine what the problem, intervention, alternative and outcome are according to the preset scenario. They are required to verify relevant evidences from the literatures, critically appraise the validity of the evidences, and apply the findings to clinical decision-making. Results of the team search and discussion were presented to judges by oral slide presentation in a well-formulated fashion relevant to the scenario. The judgment of the competition was according to an evaluation form including the quality and quantity of PICO, search skills, critical appraisal skills, application and evaluation (Additional file 1) by a team of judges. The winners and their affiliated hospitals were cited and awarded by TJCHA.

### Questionnaire design

A structured questionnaire was developed by the Division of Preventive Medicine and Health Services Research, Institute of Population Health Sciences, NHRI, Taiwan. The targets in this study were healthcare professionals participating in the EBM competitions in the years of 2009 and 2011. The questionnaires were distributed to all participants twice, first at 3 months prior to the competition and second right after the competition (Figure 1).

Questions in this survey were designed by modifying previous validated questionnaires [11,24], included items for measuring the beliefs in, knowledge of, skills in, barriers toward EBM implementation (Additional file 1). These questions were rated by Likert’s 5-point scale (strongly agree, agree, neutral, disagree, and strongly disagree). In addition, the questionnaire explored the utilization of 7 commonly-used online databases, including the Cochrane Library, MD Consult, MEDLINE/PubMed, ProQuest, UpToDate, Micromedex, and CINAHL (the Cumulative Index to Nursing & Allied Health Literature). Utilization was defined as access in recent three months by Likert’s 5-point scale (more than 12 times/month, 9–12 times/month, 5–8 times/month, 1–4 times/month, and never). The background characteristics – including gender, age, faculty position, director position, working experience, academic degree, and motivation of participation – were further examined.

Content validity was examined by 10 experts with more than 15 years of clinical experience each. The experts were asked to rate the relevance of each question item based on their expertise and to provide editorial recommendations for item improvement or elimination. Items with strong relevance were included in the final questionnaire. The internal consistency of all indexes was estimated by using Cronbach’s coefficient alpha. In this survey, the content validity index of 0.98 and Cronbach’s coefficient alpha of 0.91 indicated sufficient validity and reliability of parameters in the questionnaire.

The Ethical Review Board of the National Health Research Institutes approved the study protocol. The questionnaire was accompanied by an introductory letter stating the purpose of this study and promising confidentiality. Return of the completed questionnaire was considered as consent of participating in the study.

### Statistical analyses

The statistical analyses were conducted using a commercially available program (SPSS 12.0 for Windows, SPSS Inc., Illinois, USA). Categorical variables were analyzed using the chi-square test or Fisher’s exact test. Pair-sample *t* test was used to compare the values of means between the pretest and posttest questionnaires. Effect sizes were expressed by Cohen’s *d*[25]. Significance was defined as *p* < 0.05.

## Results

### Demographic data

The questionnaires were mailed to a total of 459 participants. Among them, 358 participants completed both pretest and posttest questionnaires, including 162 physicians, 71 nurses, 101 pharmacists, and 24 other allied healthcare professionals. The return rate of valid questionnaires was 78.0%. Participants came from 24 regional hospitals and 15 medical centers in Taiwan. Their background information is summarized in Table 2. Nurses were older and had longer working period than the other health professionals. In addition, nurses were more likely to have a director position than the others. Furthermore, pharmacists tended to have a faculty position than the other health professionals. In all of the four groups, more than 90% of health professionals rated assignment as the motive to participate in the EBM competition.

**Table 2:** Demographic data and motivation of participants

**Demography**	**All N = 358**	**Profession**			
		**Physician**	**Nurse**	**Pharmacist**	**Others**
		**N = 162**	**N = 71**	**N = 101**	**N = 24**
**Gender (%)**					
Male	161 (45.0)	125 (77.2)	3 (4.2)	28 (27.7)	5 (20.8)
Female	197 (54.4)	37 (22.8)	68 (95.8)	73 (72.3)	19 (79.2)
**Academic degree (%)**					
Under college	2 (0.6)	0 (0)	1 (1.4)	1 (1.0)	0 (0)
College*	216 (60.3)	139 (85.8)	26 (36.6)	40 (39.6)	11 (45.8)
Master’s	132 (36.9)	19 (11.7)	43 (60.6)	58 (57.4)	12 (50.0)
Doctorate	8 (2.2)	4 (2.5)	1 (1.4)	2 (2.0)	1 (4.2)
**Age (y) (± SD)**	33.2 ± 6.0	32.2 ± 5.3	36.8 ± 5.4	32.3 ± 6.2	33.5 ± 7.3
**Working period** (y)(± SD)	7.9 ± 6.1	6.0 ± 4.6	14.4 ± 5.7	6.3 ± 5.0	8.2 ± 7.0
**Director (%)**	67 (18.7)	8 (4.9)	35 (49.3)	18 (17.8)	6 (25.0)
**Faculty (%)**	111 (31.0)	32 (19.8)	23 (32.4)	50 (49.5)	6 (25.0)
**Motivation (%)**					
Assignment	328 (91.6)	148 (91.4)	65 (91.6)	93 (92.0)	22 (91.7)
Research	5 (1.4)	2 (1.2)	1 (1.4)	2 (2.0)	0 (0)
Continuing education	1 (0.3)	0 (0)	1 (1.4)	0 (0)	0 (0)
Medical accreditation	0 (0)	0 (0)	0 (0)	0 (0)	0 (0)
Interest	14 (3.9)	7 (4.3)	2 (2.8)	5 (5.0)	0 (0)
Others	10 (2.8)	5 (3.1)	2 (2.8)	1 (1.0)	2 (8.3)

* College curriculum is 7 years for medical school, 6 years for dental school and 4 years for the other professions.

### Impact of EBM competition on the belief, knowledge and skill of participants

After the competition, participants were more likely to believe that EBM is helpful in the decision-making of clinical practice than before (Cohen’s *d* = 0.156) (Table 3). Furthermore, their reported knowledge of applying EBM principles significantly increased (Cohen’s *d* = 0.489). Their reported understanding of EBM terminology – including relative risk (RR) (Cohen’s *d* = 0.235), odds ratio (OR) (Cohen’s *d* = 0.312), type I error (α error) (Cohen’s *d* = 0.361), type II error (β error) (Cohen’s *d* = 0.363), systematic review (Cohen’s *d* = 0.131), meta-analysis (Cohen’s *d* = 0.179), and number needed to treat (NNT) (Cohen’s *d* = 0.337) – also significantly increased. In addition, their reported skills in literature searching (Cohen’s *d* = 0.257), critical appraisal (Cohen’s *d* = 0.448) and clinical application (Cohen’s *d* = 0.496) significantly increased after the competition.

**Table 3:** Beliefs in, knowledge of, and skills in EBM

**Likert’s 5-point scale (± SD)**	**Before contest**	**After contest**	*p *value*
**Beliefs**			
EBM is important in the strengthening of expertise	4.16 ± 0.72	4.21 ± 0.63	0.335
EBM is useful in the improvement of patient-care quality	4.12 ± 0.74	4.18 ± 0.64	0.125
EBM is helpful in the decision-making of clinical practice	3.97 ± 0.74	4.08 ± 0.67	0.017
**Knowledge**			
My knowledge of applying EBM principles is sufficient	3.42 ± 0.71	3.77 ± 0.72	<0.001
Understanding of terminology			
Relative risk (RR)	3.38 ± 0.79	3.56 ± 0.74	<0.001
Odds ratio (OR)	3.32 ± 0.79	3.56 ± 0.75	<0.001
Confidence interval (CI)	3.58 ± 0.73	3.66 ± 0.78	0.093
Type I error (α error)	2.96 ± 0.94	3.29 ± 0.89	<0.001
Type II error (β error)	2.94 ± 0.93	3.27 ± 0.89	<0.001
Systematic review	3.62 ± 0.78	3.72 ± 0.75	0.042
Meta-analysis	3.57 ± 0.81	3.71 ± 0.75	0.002
Randomized controlled trial (RCT)	3.73 ± 0.83	3.77 ± 0.74	0.375
Number needed to treat (NNT)	3.37 ± 1.01	3.68 ± 0.82	<0.001
**Skills**			
My skill regarding the literature searching is sufficient	3.70 ± 0.64	3.87 ± 0.68	<0.001
My skill regarding the critical appraisal is sufficient	3.48 ± 0.71	3.80 ± 0.72	<0.001
My skills of applying EBM principles are sufficient	3.40 ± 0.74	3.76 ± 0.71	<0.001

* Pair-sample *t* test.

### Barriers to the implementation of EBM

Perceived barriers to the implementation of EBM are summarized in Table 4. Difficulty in critical appraisal was the most common barrier. There were significant decreases in a couple of personal barriers after the competition: literature searching (Cohen’s *d* = 0.160) and forming answerable questions (Cohen’s *d* = 0.148). In contrast, no significant change was noted in the organizational barriers, including insufficient designated personnel, lack of support from authorities and colleagues.

**Table 4:** Perceived barriers to the implementation of EBM in clinical practice

**Likert’s 5-point scale**^**† **^**(± SD)**	**Before contest**	**After contest**	*p *value*
**Personal barrier**			
Difficulty in literature searching	2.90 ± 0.83	2.77 ± 0.79	0.006
Difficulty in critical appraisal	3.08 ± 0.87	3.15 ± 0.84	0.143
Difficulty in forming answerable questions	2.62 ± 0.93	2.49 ± 0.82	0.008
Lack of basic knowledge	2.70 ± 0.91	2.64 ± 0.87	0.253
**Organizational barrier**			
Lack of designated personnel	2.70 ± 1.01	2.74 ± 0.95	0.505
Lack of support from authorities	2.63 ± 0.99	2.52 ± 0.87	0.061
Lack of support from colleagues	2.79 ± 0.96	2.83 ± 0.93	0.469

* Pair-sample *t* test.

^**†**^ Higher points represent more barriers.

### Behavior of access to the online databases

The impact of competition on participants’ searching behavior is demonstrated in Table 5. The most commonly used database was MEDLINE/PubMed, followed by UpToDate, MicroMedex, Cochrane Library, MD Consult, ProQuest, and CINAHL. During the competition period, the access rate of the following 6 online databases significantly increased – including UpToDate (Cohen’s *d* = 0.166), MicroMedex (Cohen’s *d* = 0.095), Cochrane Library (Cohen’s *d* = 0.442), MD Consult (Cohen’s *d* = 0.197), ProQuest (Cohen’s *d* = 0.225), and CINAHL (Cohen’s *d* = 0.206).

**Table 5:** Access to the online databases

**Rank**	**Likert’s 5-point scale (± SD)**	**Before contest**	**After contest**	*p *value*
1	MEDLINE/PubMed	3.46 ± 1.12	3.50 ± 1.12	0.540
2	UpToDate	3.10 ± 1.30	3.31 ± 1.23	0.001
3	MicroMedex	2.73 ± 1.49	2.87 ± 1.45	0.024
4	Cochrane Library	2.42 ± 0.90	2.85 ± 1.04	<0.001
5	MD Consult	2.21 ± 1.09	2.43 ± 1.14	<0.001
6	ProQuest	1.86 ± 1.04	2.10 ± 1.09	<0.001
7	CINAHL	1.78 ± 0.97	1.99 ± 1.07	0.001

* Pair-sample *t* test.

## Discussion

In this study, we introduced the competition as a means to disseminate the implementation of EBM. Although numerous initiatives have emerged to promote EBM [4,20,26-28], literature pertaining to examining the effectiveness of competition program is lacking. Our study is the first survey to evaluate the impact of competitions on the beliefs in, knowledge of, skills in, barriers to, and behaviors of EBM. The data demonstrate that the competition may serve as an active and useful measure to bridge the gap of evidence to practice and foster the implementation of EBM.

Educational intervention is the most common way to disseminate EBM. It can improve the knowledge of and skills in EBM. However, the improvement of behavior toward EBM is limited [4,20,29,30]. Therefore, additional methods are required in attempt to improve this step. In our study, we demonstrate that EBM competitions not only enhance the knowledge and skills but also increase the behavior of access to the online databases. These results suggest that an incorporation of competition into education could facilitate EBM learning.

In our study, the abilities to formulate a clear question, retrieve relevant literature, critical appraise study findings have improved significantly. Furthermore, the knowledge and skill of EBM implementation have increased. The competition program demonstrated benefit in helping health professionals overcoming some of the personal barriers to EBM. However, difficulty in critical appraisal although improved, but not statistically significant. It probably would take more time of practice to improve the skill of critical appraisal.

Among all the online databases that the participants have accessed, the reported usage of Cochrane Library increased the most. The Cochrane Library has been regarded as the most important resource of EBM [31]. In addition, the Cochrane Library used well-established PICO queries as a methodological standard to conduct systematic reviews [32]. Therefore it’s not surprising that our participants tended to retrieve the systematic reviews of Cochrane Library while preparing the EBM competition. In contrast, the usage of MEDLINE did not significantly increase in our study. It’s probably because MEDLINE does not sort search results based on PICO queries.

Our data provide several valuable evidences in the strategy for accelerating the dissemination of EBM implementation. First, our competition encourages team-based learning. There are a variety of attributes and dimensions that can be used to formulate a competition. Studies have supported the fact that cooperative task can achieve better learning than individual teaching [33-35]. There are an increasing number of publications showing that interactive team learning can stimulate an energetic discussion and help to consolidate learning in EBM [36,37]. Nevertheless, cooperative learning is still underutilized in postgraduate medical education. Our results support the finding that establishing multidisciplinary collaborative teamwork for competition can enhance EBM learning. Second, our findings suggest that PICO is a useful tool as a framework for the conduction of competition. The formation of a focused clinical question containing well-articulated PICO elements is one of the methods that have been suggested to search high-quality evidence efficiently [6]. Thus, mastery of skills in PICO will help health professionals in decision makings based on evidence [5,38]. Third, our competitions used validated grading system to score the results. This system offers easy direction for participants on how to prepare the skills that are required for the competition. Fourth, our competition has a preparation period of 3 months. This allows participants to learn and better understand EBM. In the meantime, it improves participants’ behaviors of EBM implementation.

This study has two strengths. First, our survey is based on the participants coming from nationwide hospitals. Second, our survey respondents in pretest and posttest were completely the same individuals. It enhanced the reliability of the questionnaire survey. With respect to limitations, this study did not include control group. Furthermore, our study is a self-report survey, not an audit of actual practice. Therefore we cannot be sure that these self-reported changes were fully translated into improved clinical care. In addition, our survey investigated the short-term effects of EBM competition. Further studies are needed to determine its long-term impact after the competition terminated.

## Conclusion

Our study has introduced a specially constructed game-based learning module for postgraduate health professionals. The EBM competition in our study is bound by rules and organized by specialists. The data indicate that applying competitions as an active learning process of EBM can help motivating the cognitive function in postgraduate education of healthcare professionals. Our data showed an improvement in relation to the implementation of EBM. The results obtained in this study suggest our nationwide structured competition model designed to inspire the learning of EBM may speed up EBM dissemination among healthcare professionals.

## Competing interests

The authors declare that they have no competing interests.

## Authors’ contribution

All authors contributed substantially to the intellectual conception of this manuscript. YHW wrote the initial draft of the paper. YWC served as the principal investigator in this work and was responsible for the study design and the drafting of the manuscript. KNK, CYY, HHL, CC, HLL, WCL contributed to the acquisition, analysis and interpretation of the data. All authors contributed to the critical revision of this draft and approved the final manuscript for publication.

## Pre-publication history

The pre-publication history for this paper can be accessed here:

http://www.biomedcentral.com/1472-6920/13/66/prepub

## Supplementary Material

Additional file 1 **Appendix I.** EBM Competition Grading Score. **Appendix II.** Questionnaire Survey for EBM Competition. Click here for file
