# Supplementary material for: Effectiveness of national evidence-based medicine competition in Taiwan
Source: BMC Med Educ. 2013 May 7;13:66. doi: 10.1186/1472-6920-13-66 (PMC3661390; doi:10.1186/1472-6920-13-66)
Supplement: Additional file 1 — Appendix I. EBM Competition Grading Score. Appendix II. Questionnaire Survey for EBM Competition. [file 1472-6920-13-66-S1.doc]

**Appendix I**

EBM Competition

Grading Score

Team name:

Judge name:

| Items | | Score | | | | |
| --- | --- | --- | --- | --- | --- | --- |
|  |  | 5 | 4 | 3 | 2 | 1 |
| PICO | 1. clearly state the problem of patient |  |  |  |  |  |
|  | 2. clearly state the intervention |  |  |  |  |  |
|  | 3. clearly state the comparison of intervention |  |  |  |  |  |
|  | 4. clearly state the measurement of outcome |  |  |  |  |  |
| Literature searching | 1. properly use keywords |  |  |  |  |  |
|  | 2. clearly state the searching strategies |  |  |  |  |  |
|  | 3. use a variety of searching methods |  |  |  |  |  |
|  | 4. enhance the searching efficiency |  |  |  |  |  |
| Critical appraisal | 1. clearly explain the reasons why these literatures are chosen |  |  |  |  |  |
|  | 2. properly use the tool guide of critical appraisal |  |  |  |  |  |
|  | 3. conscientiously appraise the reliability and benefit |  |  |  |  |  |
|  | 4. properly appraise the level of evidence |  |  |  |  |  |
| Clinical application | 1. apply on clinical decision-making |  |  |  |  |  |
|  | 2. consider cost-benefit |  |  |  |  |  |
|  | 3. compare the outcome |  |  |  |  |  |
|  | 4. state the impact of healthcare quality |  |  |  |  |  |
| Presentation | 1. valid content |  |  |  |  |  |
|  | 2. easy-reading slides |  |  |  |  |  |
|  | 3. performance of presenter |  |  |  |  |  |
|  | 4. well-controlled time |  |  |  |  |  |
| Comment: | | | | | | |

**Appendix II**

Questionnaire Survey for EBM Competition

National Health Research Institutes

1. Personal Information

Gender: □ male □ female

Birth year: ________

Your profession: □ doctor □ nurse □ pharmacist □ others

Are you currently a faculty? □ yes □ no

Are you currently a director in clinical service? □ yes □ no

Your total working period after obtaining license: ______ year(s)

Academic degree □ under College □ College □ master □ Ph. D.

The major motivation of participation in this EBM competition:

□ assignment

□ research

□ continuing education

□ medical accreditation

□ interest

□ others

1. Perceptions of EBM
   1. Do you agree the following statement?

|  | strongly agree | agree | neutral | disagree | strongly disagree |
| --- | --- | --- | --- | --- | --- |
| EBM is important in the strengthening of expertise | □ | □ | □ | □ | □ |
| EBM is helpful in the decision-making of clinical practice | □ | □ | □ | □ | □ |
| My knowledge of applying EBM principles is sufficient | □ | □ | □ | □ | □ |
| My skill regarding the literature searching is sufficient | □ | □ | □ | □ | □ |
| My skill regarding the critical appraisal is sufficient | □ | □ | □ | □ | □ |
| My skills of applying EBM principles are sufficient | □ | □ | □ | □ | □ |

- 1. Do you agree you understand the following term?

Explanation: strongly agree – understand and could explain to others agree –understand somewhat

neutral –understand but could not explain to others

disagree – do not understand but hear of it

strongly disagree – never hear of it

|  | strongly agree | agree | neutral | disagree | strongly disagree |
| --- | --- | --- | --- | --- | --- |
| Relative risk (RR) | □ | □ | □ | □ | □ |
| Odds ratio (OR) | □ | □ | □ | □ | □ |
| Confidence interval (CI) | □ | □ | □ | □ | □ |
| Type I error (α error) | □ | □ | □ | □ | □ |
| Type II error (β error) | □ | □ | □ | □ | □ |
| Systematic review | □ | □ | □ | □ | □ |
| Meta-analysis | □ | □ | □ | □ | □ |
| Randomized controlled trial (RCT) | □ | □ | □ | □ | □ |
| Number needed to treat (NNT) | □ | □ | □ | □ | □ |

- 1. Do you agree the following statement is your barrier to apply EBM principles?

|  | strongly agree | agree | neutral | disagree | strongly disagree |
| --- | --- | --- | --- | --- | --- |
| Difficulty in literature searching | □ | □ | □ | □ | □ |
| Difficulty in critical appraisal | □ | □ | □ | □ | □ |
| Difficulty in forming answerable questions | □ | □ | □ | □ | □ |
| Lack of basic knowledge | □ | □ | □ | □ | □ |
| Lack of designated personnel | □ | □ | □ | □ | □ |
| Lack of support from authorities | □ | □ | □ | □ | □ |
| Lack of support from colleagues | □ | □ | □ | □ | □ |

- 1. How often have you accessed the following database during the past 3 months?

average times/month

>12 9-12 5-8 1-4 0

CINAHL □ □ □ □ □

Cochrane Library □ □ □ □ □

MD Consult □ □ □ □ □

MEDLINE □ □ □ □ □

ProQuest □ □ □ □ □

UpToDate □ □ □ □ □

MicroMedex □ □ □ □ □
